# Skin Cancer in the Rural South: Strengthening Dermatologic Training for Frontline Primary Care

**DOI:** 10.7759/cureus.111042

**Published:** 2026-06-17

**Authors:** Sarah E Burstein, Howard I Maibach

**Affiliations:** 1 Brody School of Medicine, East Carolina University, Greenville, USA; 2 Department of Dermatology, University of California San Francisco School of Medicine, San Francisco, USA

**Keywords:** dermatology, dermatology education, healthcare disparities, health policy, medical education, primary care, primary care education, rural health disparities

## Abstract

Skin cancer remains a major public health challenge in the rural Southeastern United States (US), where elevated disease burden coincides with limited access to dermatologic care. Rural populations face increased risk due to occupational exposure to ultraviolet light, poverty, lower rates of routine skin screening, and transportation barriers, yet many nonmetropolitan counties have no practicing dermatologists. Consequently, primary care physicians (PCPs) often serve as the first, and sometimes only, clinicians evaluating suspicious skin lesions. Despite this responsibility, dermatology remains underrepresented in undergraduate medical education and primary care training. Because dermatologic diagnosis relies heavily on pattern recognition, morphologic assessment, and familiarity with presentations across diverse skin tones, insufficient training may contribute to delayed recognition of premalignant and malignant lesions, particularly among medically underserved populations. This editorial argues that strengthening dermatologic education within general medical training is a pragmatic and immediately actionable strategy to address workforce maldistribution and reduce rural skin cancer disparities. This is not a proposal for PCPs to replace dermatologists, but rather a call to equip frontline clinicians with the foundational skills needed to recognize, triage, and appropriately manage high-risk dermatologic presentations.

## Editorial

In the rural Southeastern United States (US), skin cancer is common, but dermatologists are not.

Nonmelanoma and melanoma skin cancers are among the most prevalent malignancies nationwide [1]. In the rural Southeast (defined as the rural regions of North Carolina, South Carolina, Georgia, Alabama, Mississippi, Louisiana, Arkansas, Tennessee, Kentucky, Virginia, and West Virginia), the burden is especially high. Indeed, skin cancer incidence is particularly elevated in rural parts of these states compared to their urban counterparts, with delayed diagnoses and more advanced disease at presentation [2,3]. These challenges are compounded by broader geographic and socioeconomic disparities in skin cancer outcomes. For example, a recent national analysis found that lower sociodemographic index (SDI) states experienced significantly higher melanoma mortality (2.23 versus 1.86 per 100,000 population) and disability-adjusted life-year burdens (69.6 versus 57.0 per 100,000 population) than higher-SDI states despite comparable incidence rates, suggesting persistent inequities in early detection and access to care [4].

This public health challenge is magnified by a stark geographic mismatch: while over 20% of the US population lives in rural areas, only about 10% of physicians, and an even smaller fraction of dermatologists, practice there [5,6]. In many nonmetropolitan counties in the rural South, there are zero practicing dermatologists [6,7]. Such a workforce shortage frequently overlaps with communities experiencing greater cumulative ultraviolet light exposure from outdoor occupations, higher rates of poverty, less insurance coverage, and greater geographic isolation, compounding barriers to timely dermatologic evaluation and treatment [2,4,7].

In the absence of readily available dermatologic care, primary care physicians (PCPs) in this region often serve as the first, and only, medical contact for patients with suspicious or evolving skin lesions. However, most PCPs receive minimal formal education in dermatology, typically confined to a handful of lectures and lacking mandatory clinical exposure [8,9]. A 2015 national study found that nearly 88% of medical students viewed their dermatologic training as insufficient, as demonstrated by an average score of just 46.6% on standardized dermatology assessments among graduating medical students [8]. More recent investigations confirm that dermatology remains underrepresented in the curricula of many US medical schools, with clinical exposure either optional or absent altogether [9].

The mismatch between clinical responsibility and educational preparation may have important consequences for patient care. Dermatologic diagnosis relies heavily on pattern recognition, morphologic nuance, and familiarity with presentations across different skin tones. Insufficient training can lead to missed or delayed diagnoses of malignancy, particularly among medically underserved patients with limited access to follow-up care. Melanoma may be mistaken for a benign melanocytic nevus; cutaneous squamous cell carcinoma (SCC) may be dismissed as inflammatory dermatoses; actinic keratoses (AK) may go unnoticed entirely until disease progression occurs [1].

The implications of this are profound. Melanoma accounts for the majority of skin cancer-related deaths and requires early identification to optimize survival outcomes [4,10]. SCC, although less fatal, carries significant morbidity and metastatic potential if not promptly diagnosed and treated [1,10]. Basal cell carcinoma, while less aggressive, can still lead to disfigurement or local invasion [1,10].

As a medical student at a university committed to training PCPs serving the rural and medically underserved in the Southeast US, I find it concerning that dermatology remains such a peripheral component of general medical education. This educational gap risks perpetuating health disparities, particularly for patients where high skin cancer incidence intersects with poverty, low health literacy, and transportation barriers.

Because expanding the dermatology workforce in rural areas will require time and substantial resources, strengthening the dermatologic competency of frontline PCPs may represent one of the most immediately actionable strategies for improving skin cancer detection. Addressing this requires more than recognizing the dermatologist workforce shortage; it necessitates targeted reforms in medical education. Medical schools and primary care training programs should consider incorporating mandatory dermatology exposure focused on common and high-risk conditions frequently encountered in frontline practice. Educational strategies may include structured teaching in lesion morphology, skin cancer recognition, and dermatologic evaluation across diverse skin tones, supplemented by case-based learning and supervised clinical experiences when feasible. Teledermatology and digital image-based instruction may further expand educational access, particularly at institutions and training sites lacking dedicated dermatology departments, although challenges related to broadband access, reimbursement, and technological infrastructure may limit implementation in some rural settings.

Practical curricular reform need not require extensive restructuring of existing medical education programs. Even brief longitudinal exposure to dermatology through integrated skin examination teaching, teledermatology case conferences, dedicated lesion recognition modules, and required clinical experiences may improve diagnostic confidence among future PCPs. Partnerships between dermatologists and primary care educators could further support scalable teaching models at institutions without home dermatology departments or residency programs.

This is not a proposal for PCPs to assume the full role of dermatologists. Rather, it is a pragmatic call to strengthen dermatologic training within general medical education so that PCPs can confidently recognize, triage, and manage premalignant and high-risk dermatologic presentations. Dermatology must be reclassified not as a subspecialty afterthought but as a foundational skill set for primary care to significantly reduce diagnostic delays and improve patient outcomes in dermatology-scarce regions such as those in the rural South.

## References

[1] Caraviello C, Nazzaro G, Tavoletti G (2025). Melanoma skin cancer: a comprehensive review of current knowledge. Cancers (Basel).

[2] Zahnd WE, Fogleman AJ, Jenkins WD (2018). Rural-urban disparities in stage of diagnosis among cancers with preventive opportunities. Am J Prev Med.

[3] Semprini J, Gadag K, Williams G, Muldrow A, Zahnd WE (2024). Rural-urban cancer incidence and trends in the United States, 2000 to 2019. Cancer Epidemiol Biomarkers Prev.

[4] Akbarialiabad H, Leachman SA, Taghrir MH (2026). Melanoma in the United States, 2010-2021: national trends and state-level patterns. Arch Dermatol Res.

[5] (2017). US Government Accountability Office: Physician workforce: Locations and types of graduate training were largely unchanged, and federal efforts may not be sufficient to meet needs. https://collections.nlm.nih.gov/catalog/nlm.

[6] Feng H, Berk-Krauss J, Feng PW, Stein JA (2018). Comparison of dermatologist density between urban and rural counties in the United States. JAMA Dermatol.

[7] Vaidya T, Zubritsky L, Alikhan A, Housholder A (2018). Socioeconomic and geographic barriers to dermatology care in urban and rural US populations. J Am Acad Dermatol.

[8] Ulman CA, Binder SB, Borges NJ (2015). Assessment of medical students' proficiency in dermatology: are medical students adequately prepared to diagnose and treat common dermatologic conditions in the United States?. J Educ Eval Health Prof.

[9] Shadid AM, Albdaya NA, Aldosari MS, Habib M, Aldera RM, Almohaimeed DH, Altalhab S (2023). Dermatology core curriculum in medical school and its association in the selection of dermatology as a future career: a nationwide cross-sectional study. J Dermatol Dermatol Surg.

[10] Sathe NC, Zito PM (2026). Skin cancer. StatPearls.

